# Monitoring, reporting and regulating medicine quality: tensions between theory and practice in Tanzania

**DOI:** 10.1136/bmjgh-2020-003043

**Published:** 2021-05-28

**Authors:** Heather Hamill, Elizabeth David-Barrett, Joseph Rogathe Mwanga, Gerry Mshana, Kate Hampshire

**Affiliations:** 1 Department of Sociology, University of Oxford, Oxford, UK; 2 School of Law, Politics and Sociology, University of Sussex, Brighton, UK; 3 Epidemiology, Biostatistics, and Behavioural Sciences School of Public Health, Catholic University of Health and Allied Sciences Bugando, Mwanza, United Republic of Tanzania; 4 Mwanza Research Centre, National Institute for Medical Research, Mwanza, United Republic of Tanzania; 5 Department of Anthropology, Durham University, Durham, UK

**Keywords:** health policy, health systems, public health, qualitative study

## Abstract

In 2012, the WHO launched its Global Surveillance and Monitoring System (GSMS) for substandard and falsified medicines, with the aim of improving the quality of reporting and using the data to inform post-market surveillance and build regulatory capacity. However, from a regulatory governance perspective, its effectiveness depends on the willingness and ability of actors ‘on the ground’ to identify, report and investigate possible infringements and to enforce penalties. This paper presents findings from 27 interviews with representatives of agencies charged with regulating pharmaceutical markets and 4 interviews with pharmaceutical industry representatives in Tanzania. Their experiences provide important insights into how the theorised mechanism between reporting and a reduction in undesirable behaviours can play out in a low-income context, revealing hidden assumptions about regulator behaviour and motivations. A combination of chronic under-resourcing, information gaps and enforcement challenges conspires to limit the efforts of local regulators to achieve the GSMS goals, shedding new light on the relationship between apparent ‘misconduct’ and structural constraints.

**Key questions**

**What is already known?**

- The WHO’s Global Surveillance and Monitoring System (GSMS) aims to improve the quality of reporting of substandard and falsified (SF) medicines, inform post-market surveillance and build regulatory capacity.
- Regulatory effectiveness depends on the willingness and ability of actors ‘on the ground’ to identify, report and investigate infringements and to enforce penalties.

**What are the new findings?**

- ‘On the ground’ regulators often lack resources to fulfil their roles properly.
- Regulators’ ability and willingness to investigate and sanction allegations is inhibited by difficulties in cooperating with other agencies in order to collect evidence.
- Regulatory violations are often ignored and, even if exposed, are unlikely to be sanctioned.

**What do the new findings imply?**

- The GSMS mechanism is severely inhibited by capacity constraints, economic and social barriers to reporting and poor diagnostic tools.
- Our findings cast doubt on the extent to which weak regulatory capacity of SF medicines is a problem of ‘misconduct’.

## Introduction

Substandard and falsified (SF) medicines constitute a major public health threat, particularly in low-income and middle-income countries (LMICs), where regulatory capacity may be relatively weak, disease burdens high and where reliance on imported medicines and supplies results in complex and opaque supply chains.^1 2^ The WHO^3^ recently estimated over 10% of pharmaceutical products globally are of poor quality, whether through deliberate fraud, poor manufacturing practice or deterioration. Poor-quality antibiotics and antimalarials are believed to result in more than 200 000 child deaths per year^1 3–5^ and contribute significantly to antimicrobial resistance.^6 7^

As early as 1999, the WHO outlined factors facilitating the manufacture and trade in poor-quality medicines and recommended remedial measures to address access, governance and technical capacity.^8^ At the heart of the current WHO strategy is the Global Surveillance and Monitoring System (GSMS). The GSMS, established in 2012, aims to ‘work with WHO Member States to improve the quality of reporting of SF medical products, and, importantly, to ensure the data collected are analysed and used to influence policy, procedure and processes to protect public health, at the national, regional and the global level’.^5^ The system receives, collates and responds to reports from focal points in national medicine regulatory authorities, inspectorates, enforcement units, pharmacovigilance centres and quality control laboratories. The short-term goal is to facilitate emergency responses to SF medicines in circulation, with a longer-term aim of using the data to inform post-market surveillance and build regulatory capacity. This article focuses on these longer-term aims, using a regulatory governance theoretical framework and providing empirical evidence from the ‘on-the-ground’ experiences of regulators in a lower/middle-income setting, Tanzania.

The GSMS is a form of regulatory governance concerned with standard-setting and behaviour modification.^9–11^ Through gathering and collating information, it aims to reduce the harm caused by poor-quality medicines through changing the behaviour of actors involved in the manufacture, trade and oversight of pharmaceuticals. Evidence suggests that monitoring and reporting mechanisms can improve governance and provision of public services^12^ including in healthcare systems in LMICs.^13 14^ They are used in various settings with the aim of reducing misconduct, crime and corruption, either by activating improved social accountability and reducing asymmetric information in ‘principal-agent’ situations^15 16^ or by motivating governments and regulators to improve compliance, in order to boost their reputation vis-a-vis peers.^17 18^ In the current context, the aim of reporting mechanisms is twofold: to *detect* behaviours responsible for the circulation of SF medicines and to *deter* them in future; sometimes called ‘direct’ and ‘indirect’ effects.^19 20^ However, the pathway from collecting data to changing behaviours is not straightforward and depends on actors being willing and able to comply with rules through several stages.

**Figure 1:**
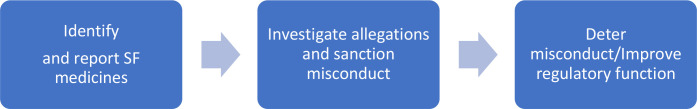
Necessary stages for a monitoring and reporting system to lead to improved governance. SF, substandard and falsified.

In the case of the GSMS, the necessary chain of events is as follows. First, SF medicines entering the market must be identified, either by regulators or by others reporting suspicions to regulators. Second, regulators must act on the information: undertaking investigations, imposing sanctions on wrongdoers and fixing any regulatory weakness. This leads to the third step, whereby behaviours change, limiting the opportunities for poor-quality medicines to penetrate and circulate in the market (see figure 1).

If it works effectively, such a system could reduce the overall prevalence of SF medicines in circulation through multiple mechanisms: ensuring poor-quality products on the market are detected and removed; improving regulation to prevent SF medicines from reaching the market in the first place; and altering the cost-benefit calculus of undertaking and/or reporting dubious practices (if perceptions change about the likelihood of detection and effective sanction). However, the functioning of the GSMS and its capacity to achieve its aims depends on the very earliest stages of the process: the identification and reporting of suspect products. It therefore relies heavily on national regulators having good access to information and, crucially, *being willing to report it*. National regulators depend largely on receiving reports from multiple local actors, including regional and district representatives of Food and Drugs Authorities, Pharmacy Councils, Standards Authorities and others.

The purpose of this study is to understand how regulators working ‘on the ground’ in Tanzania (a low-income country with relatively well-functioning regulatory authorities^21^) manage the various steps of the monitoring and reporting system set out above. We present data, from interviews with 27 national-level and local-level regulators and 4 interviews with industry representatives of local pharmaceutical companies, that show how capacity constraints, institutional arrangements, economic incentives and ethical dilemmas intersect in the ‘real world’, producing serious barriers to movement through this chain of action. We argue that these obstacles are likely to prevail in many LMIC contexts, creating significant challenges for effective quality assurance.

### Tanzanian context

Pharmaceutical medicines in Tanzania can legally be sold in licensed pharmacies and in Accredited Drug Dispensing Outlets (ADDOs), the latter coming from a donor-supported initiative launched in 2003 by the (then) Tanzania Food and Drugs Authority (TFDA) to improve access to essential medicines for under-served rural populations.^22^ Pharmacies can sell all three categories of retailed medicines (prescription only, pharmacy only and general sales list), while ADDOs are only permitted to retail those on the general sales list. In May 2020 there were 1775 registered pharmacies in Tanzania (https//www.pc.go.tz/premises/all.php). In 2019, there were 14 036 accredited ADDOs in Tanzania (Tanzania Pharmacy Council, 2020) known in Swahili as ‘Maduka ya dawa muhimu’ (literally, essential medicines shop). In addition, a proportion of pharmaceutical medicines are sold illegally from unregulated outlets including grocery and other unlicensed shops, market stalls and itinerant peddlers selling from car boots or door-to-door.

In Tanzania, the task of regulating pharmaceutical licencing and quality is distributed across multiple agencies. During our fieldwork, the TFDA was the federal agency with primary responsibility for ensuring the quality, safety and efficacy of medicines, medical devices and diagnostics, overseeing production, import, distribution and sales (these responsibilities were re-designated in 2019 to the newly formed Tanzania Medicines and Medical Devices Authority, TMDA). Complementing the work of the TFDA/TMDA is the Pharmacy Council of Tanzania, which oversees, regulates and controls professional conduct in retail pharmacies. Other federal agencies implicated in the regulation and governance of pharmaceuticals in Tanzania include (inter alia) the Tanzania Revenue Authority, Fair Competition Commission, Tanzania Police Force, Environmental Protection Agencies and Trading Officers.

The TFDA/TMDA is considered to be one of the most effective medicine regulatory authorities in sub-Saharan Africa, and Tanzania is the first African state to be recognised by the WHO as having achieved a well-functioning, regulatory system for medical products.^23^ Nonetheless, regulatory authorities in Tanzania share some of the constraints experienced in other LMICs, including chronic under-resourcing, weak infrastructures and excessive workloads resulting from a high turnover in products.^21^ As we discuss later, these have serious implications for the efficacy and sustainability of processes to protect the quality of medicines reaching consumers.

## Methods

This study adopted a qualitative, interpretative methodology, based on semi-structured interviews designed to capture participants’ experiences and interpretations on their own terms, while ensuring that key pre-specified themes were covered systematically. Over two periods of fieldwork in Tanzania (June 2016 and January 2017), the authors (academic social scientists based in Tanzania and the UK) conducted 27 interviews with regulators representing 6 national level and 10 regional and district level authorities regulating medicines and medical products, and licensing pharmacies and ADDOs. Four additional interviews were conducted with representatives of the local pharmaceutical industry in Tanzania. Interviews were conducted in two main locations: Dar-es-Salaam (the main commercial and import hub of Tanzania) and Mwanza Region (situated in northwest Tanzania, on the southern shores of Lake Victoria). Within Mwanza Region, we worked principally in the regional capital, Mwanza City, and the smaller settlements of Magu and Misungwi districts.

The 27 regulators were selected purposively to achieve a maximum variation sample. Selection was based on their positions within key agencies responsible for pharmaceutical regulation at national and regional/district levels, with data collection continuing to the point of theoretical saturation. Interview topics included questions about agencies’ regulatory responsibilities, individual roles and the daily challenges individuals faced in fulfilling their statutory duties. The four pharmaceutical industry representatives were selected purposively for their expert local knowledge and interviews focused on gaining an overview of the industry, its evolution and future development. Interviewers took hand-written notes rather than audio recordings, because of the potential sensitivity of the material. Notes were subsequently typed up and analysed following the principles of Grounded Theory, whereby theoretical insight emerges from data through an iterative process of close-reading, coding and testing of nascent hypotheses through subsequent fieldwork.^24 25^ In the first cycle, data were coded descriptively according to the stage of the regulatory process. In the second cycle, codes were analysed thematically to identify motivations for, and obstacles to, reporting, investigating and so on. The quotations throughout this paper are illustrative of experiences reported by the wider sample.

Because of the sensitive nature of some information presented, we have taken great care to protect interviewees’ anonymity, by not giving any names or other potentially identifying information, instead identifying them with more general categories (eg, national-level regulator). In this study we have followed Standards for Reporting Qualitative Research reporting guidelines.^25^

### Patient and public involvement statement

The study was designed in collaboration with attendees at a Stakeholder meeting for national-level regulators in Dar-es-Salaam in May 2016. The stakeholder group provided input into the research design, the research instruments and the sampling methods.

## Results

### Perceptions of the SF medicine problem

An important precursor to engagement with reporting/monitoring systems like GSMS is recognition of the ‘problem’. Our fieldwork in Tanzania suggested there was little shared understanding among regulators about the scale, nature and source of SF medicines in the country.

In relation to scale and severity, some interviewees *reported* the prevalence of SF medicines to be high, representing a major and systemic threat to public health (‘Trust me’, said one local regulator in Mwanza City, ‘It is a very big problem’), while others downplayed the risks. For some, the major threat came from large-scale operations manufacturing and/or importing substantial quantities of illegal products, while others suggested that most poor-quality products came from small-scale, ‘lower tech’ outfits; for example, generic medicines being repackaged at a very local level. ‘They are looking for cheap money by trying to get more money for generic drugs. This is a very small-scale practice done by people in the streets who might just make ten labels and that is it. Not done by professionals’, said one local regulator. Another gave us this example:

In Tanzania, we have captured some citizens filling amoxicillin capsules with flour. Also, people use machines to alter expiry dates. These are just cheap labelling machines so easy and cheap to do—small scale operations.

There was also variation in perceptions about the precise nature of the problem, echoing debates in the academic literature on SF medicines (eg, Hamilton *et al*^26^) and also in Kingori and Gerrets’ (p380) discussion of pseudo global heath: ‘the indeterminate, blurry and messy spectrum that exists between binary oppositions such as fake/real or authentic/inauthentic’.^27^ The two interview excerpts above describe deliberate falsification but with different possible effects on the user. In the first case (passing off a generic product as a more expensive branded version to increase profits) the end result *may* still be of high quality, although of uncertain and unverifiable source. In the second case (capsules filled with flour rather than antibiotic), the result is clearly unlikely to have any therapeutic effect beyond a placebo. Likewise, fraudulent manipulation of expiry dates is likely to impact negatively on the quality of the product, but the severity will depend on the stability of the molecules and the conditions of storage.

Other interviewees stated that sub-standard production (resulting in products that have been authorised by national regulatory authorities but are nonetheless non-compliant with quality standards) or post-manufacture deterioration (eg, resulting from inadequate transport or storage facilities) were bigger threats than deliberate falsification (fraudulent misrepresentation of a drug’s identity, composition or source). However, as one national-level regulator noted, there can be a fine line between deliberate malpractice and strategic business decisions to under-invest in quality assurance. Regulators were also concerned about medicines registered in a neighbouring country but not licenced for sale in Tanzania; here, the issue was not necessarily about quality per se, but rather the integrity and governance of Tanzania’s pharmaceutical markets.

Finally, opinions differed as to the origin of SF medicines and point of penetration into Tanzanian supply chains. Some, like this national government representative, raised doubts about quality control in domestic production:

Many of them [local manufacturers] don’t have good quality control. For example, the issue of not putting the right number of tablets in the bottle. This indicates poor manufacturing practice and there may be other quality issues too.

However, most blamed the large number of foreign imports, especially those coming from India, China and Nigeria. There was general consensus that Tanzania’s ‘porous borders’ made it particularly vulnerable:

It is a global issue—you cannot say you are safe. We have porous borders with neighbouring countries […]. In Tanzania we have good regulation, but we have a very long coastline and many unofficial ports where goods can enter. Or they may come across land borders from DRC, Burundi, Uganda, Kenya, Malawi … You cannot build a wall! (National-level regulator)

In Mwanza City, reported traffic across Lake Victoria made it particularly difficult to confirm the product’s country of origin:

They come to Tanzania from other countries around the Lake: Kenya, Uganda, Rwanda, Burundi, DRC. But you can’t actually tell where they are manufactured. They might just pass through these countries. (Local regulator, Mwanza City)

Questions about the relative importance of formal and informal entry points also elicited mixed opinions. Several interviewees in Dar-es-Salaam suggested that regulation at official ports of entry was so weak that it made no sense to avoid them. Instead of making the effort to smuggle SF medicines through a remote or ‘informal’ channel, said one, one could go straight through the main Dar-es-Salaam ports, with very little chance of being checked.

Against this background of uncertainty and variation in perception of the nature, scale and severity of the SF ‘problem’, we consider in turn each of the three stages needed for an effective monitoring and reporting system to improve pharmaceutical governance (outlined in the Introduction section), noting constraints experienced by actors at each point.

### Identifying and reporting suspect products

The first requirement of a well-functioning monitoring and reporting system is people’s ability and willingness to identify and report suspect products. Our interviewees saw this unequivocally to be a vital public health responsibility. Many applauded efforts of organisations like the TF(M)DA, telling us: ‘the TFDA is one of the best in Africa. They are thoroughly examining documents, so I am very confident that whatever comes into the country is good’ (National-level industry representative). However, those working ‘on the ground’ highlighted various factors that conspired to frustrate their efforts.

First, in common with many LMICs, regulatory agencies in Tanzania (especially at local level) are often under-resourced; their capacity outstripped by the enormity of the task. During our fieldwork, the TFDA Zonal Office in Mwanza, responsible for post-market surveillance in the Lake Zone (an area of 120 000 km^2^, half the size of the UK, with a population of over 12 million), had just four medicine inspectors. Another regulator told us that, in Mwanza City alone, there were more than 80 licenced pharmacies, 500 ADDOs and 1000 dispensaries. With minimal staff, it was impossible to cover all of these, let alone moving outside of the city, tracking down informal sellers or patrolling the vast lake shore.

Human resource constraints are further compounded by poor transport infrastructure and a lack of vehicles, making travel to more remote locations costly and time-consuming. By their own admission, regulators tended to focus their efforts on easily reached locations—a manifestation of ‘tarmac bias’.^28^ As one local regulator put it, ‘They [inspectors] don’t go into the interior. In rural areas, the people haven’t even heard of [regulatory agency]’. In other situations, serious deficiencies in regulatory capacity have been shown to lead to poor enforcement and can create systems vulnerable to corruption and bias; for example, in cases where businesses ‘facilitate’ inspectors’ visits by covering travel expenses and providing accommodation.^29 30^

A second key constraint was a lack of reliable information and expertise required to distinguish a poor-quality medicine from a good one. In rural areas, inspections are often carried out by Trade Officers, with no pharmacological training or qualifications, whose work is limited to checking paperwork and conducting basic visual inspections. As one commented:

Understanding medicines needs a professional. I’m a professional in business, not medicine. I may fail to detect that this medicine has expired or is not the proper one. I may get the feeling that something is wrong, but I can’t pin it on anything because I don’t know enough. The only thing I have to go on is the license. (Regional Trade Officer)

In reality, it is almost impossible even for a well-qualified pharmacist to detect a carefully manufactured and professionally packaged fake. While the WHO have invested in bolstering capacity in chemical analysis, the vast majority of inspectors have no access to even the most basic laboratory equipment. Developments in low-cost technologies like the WHO-endorsed Global Pharma Health Fund Minilab have potential to bridge this important information gap. However, to date, they are still not widely available to regional/local authorities in Tanzania and elsewhere. Moreover, the sensitivity and reliability of low-cost technologies for detecting SF medicines have been questioned,^31^ while higher-specificity techniques like high-performance liquid chromatography remain prohibitively expensive in most LMIC settings. Instead, regulators rely on visual inspection and ‘hunches’.

Pharmacovigilance also depends on patients, pharmacists and healthcare professionals reporting ‘adverse events’, including medicines not having the expected therapeutic effect. However, a combination of low diagnostic capacity, widespread polypharmacy and the possibility of multiple comorbidities make it almost impossible to ascertain the precise therapeutic effects of a particular medicine or likely causes of treatment failure (eg, poor medicine quality vs incorrect diagnosis). As one interviewee explained:

The main problem is polypharmacy: people taking multiple medicines so they cannot tell what has cured them. There are just too many uncontrolled drugs in circulation. […] People are treating diseases that are not there. They are consuming a lot of medicine unnecessarily because of poor regulatory mechanisms. (District Medical Officer)

Third, even with adequate information and resourcing, there may be strong economic disincentives to reporting suspect products. Retailers may not voice suspicions about products because of the reputational damage and consequent loss of business that may follow. As we have reported elsewhere,^32 33^ in these contexts of fierce competition, risking a hard-won reputation for selling trusted medicines by reporting cases of apparently poor efficacy rarely makes business sense. Furthermore, according to our interviewees, when suspicions are reported, shop-owners have to pay 25% of product recall costs. For small-scale businesses, this represents a serious immediate financial risk on top of possible longer-term reputational damage.

Finally, reporting can carry risks to personal safety. Several regulators received information (tip-offs) from so-called ‘well-wishers’, ranging from health-workers, to informants with information on criminal organisations, to unhappy consumers. Reporting to protect others from harm, thereby serving the public interest, is a form of ‘whistle-blowing’. However, even in high-income countries, we know there are many serious disincentives to whistle-blowers, who often face retribution and ostracism.^34 35^ Our interviews confirmed that reporting SF medicines can incur considerable costs for informants, who may also be required to assist in further investigations, increasing their risks of exposure. As one local regulator put it, ‘These tip-offs are anonymous because these business-people have money and are powerful; the well-wishers don’t want to be identified’.

Indeed, information in Tanzania is more often shared in the opposite direction, with knowledge of the arrival of inspectors spreading quickly throughout a community. Another local regulator explained:

There are general stores that sell a few medicines. They are not allowed at all to do this … but once you reach the village information goes quickly so everyone will either hide the medicines or close the shop, so you won’t realise.

### Investigating allegations and sanctioning misconduct

The next stage for a regulator is investigation and, where appropriate, sanctioning those involved in misconduct. Investigating allegations often entails inter-agency collaboration, and interviewees reported many examples of regulators working together to tackle a problem, sometimes involving police and other enforcement agencies. However, inter-agency working is not always straightforward: operational disagreements about the focus and execution of investigations, along with the ever-present risk that information will be leaked ahead of time about an imminent inspection, was often said to hinder the progression of cases.

Like whistleblowing, investigating allegations of irregular or illegal practice can carry personal risks for regulators. One local regulator recalled violent confrontations resulting from ‘under-cover work’, giving the example of a counterfeit labelling operation he had been investigating and noting that routine inspections could start peacefully but end violently. Another local regulator said bluntly, ‘If you are not friendly to them [shopkeepers], you may find your life becoming shorter’. Often working on their own, or in very small teams, these concerns may make regulators more cautious about investigating non-compliance and enforcing regulations.

Ethical dilemmas can arise when carrying out inspections on the ground. In rural areas, where medicine outlets are scarce, there may be serious implications of closing down the only ADDO serving a large population: ‘People need medicine. If you close the shop, it is not just the owner who will suffer, the whole village will suffer’. This regional regulator told us that, in such cases, they may decide to work with retailers to help them comply with regulations rather than acting punitively and cutting off access to essential medicines for a whole community: ‘So, you don’t close the shop; you counsel the man that he has to pay and follow the government regulations’. However, this is not always straightforward:

On the first day you counsel him then you have to become as if furious and close the door. He will tell the reality—I don’t have money I will pay [the licensing fee] later but the next day he doesn’t come and he doesn’t come the day after that so after two weeks you go back again but if he doesn’t pay that doesn’t mean you close the shop.

### Deterring misconduct and improving regulatory function

The final stage in our accountability chain is based on optimal deterrence theory and postulates that individuals will desist from engaging in misconduct—such as failing to comply with regulations or knowingly supplying substandard medicines—if they assess that risks of being caught and punished outweigh the benefits.^36^ Thus, for the GSMS to lead to effective deterrence, it must be perceived to have increased the risks and penalties associated with trading and selling SF medicines.

There is little current evidence that regulators are perceived as tough enforcers; the vast majority of infringements appear to go unpunished. Most interviewees struggled to recollect any cases of successful prosecutions. One Mwanza regulator could recall only one case, in 2012, involving a local manufacturer; however, after 4 years, the case was still in the courts. Another cited a single instance of a pharmacy being closed down (for stocking US$45 000 worth of unregistered drugs). Even successful prosecutions reportedly resulted in weak penalties. A local regulator complained that the maximum penalties were a fine of 5 million TZS (approximately 1750 GBP) or 3 years imprisonment but ‘because there is no minimum penalty, they can usually get away with a lot less’. In practice, within Mwanza at least, penalties apparently rarely exceeded closing a shop down for a few weeks.

Other interviewees spoke only in very general terms about enforcement and penalties, implying that current reporting mechanisms were failing to deter those violating regulations. ‘In Tanzania we have very good policy—the issue is implementation’, said one local regulator, reflecting a commonly held view. Another told us, ‘There are laws, but enforcement is weak. Small shops will sell you two capsules of amoxicillin. There are laws that prohibit this, but it happens’.

Some interviewees spoke about the possibility of corruption. One national-level regulator suggested ‘some of them [inspectors] might be more interested in collecting fees [bribes] than in controlling the goods’, while another claimed police officers might be ‘persuaded’ to modify documents before a case came to court. One local industry representative pointed to a more systemic problem of political interference and weak rule of law:

It depends on the head of state and the ministers. The problem is corruption. So, if the TFDA inspectors find a problem and sue you, people go through the politicians. Politicians put pressure on law enforcers to drop the case because they are party supporters and the politicians need their votes. Politicians should enforce the laws regardless of their party.

Overall, the evidence presented here suggests it is unlikely that the current monitoring and reporting regime is having any significant deterrent effect.

## Discussion

There is a growing body of research and policy directives on the importance of reporting SF medicines. This builds on theory and empirical evidence suggesting that transparency and reporting can enhance accountability and improve provision of public and private goods and services. However, much empirical evidence about this relationship is based on studying the effects of introducing transparency in high-income contexts; even in these contexts evidence suggests that a strong accountability ecosystem is necessary to achieve the best results.

The interview data presented earlier provide a glimpse into the lived experiences of regulators in Tanzania who work on the ground to enact policy edicts from the WHO, national government and others in a complicated and often messy ‘real world’. Their experiences provide important insights into how theorised mechanisms between reporting and a reduction in undesirable behaviours play out in a low-income context, helping to identify many hidden assumptions about the behaviour of regulators vis-a-vis commercial actors and consumers of medicine.

First, we find that regulators often lack resources to fulfil their roles properly, particularly given the need to travel long distances in a country with poor infrastructure. This lack of capacity could, in theory, be compensated by a healthy reporting system, if other stakeholders with an interest in improving accountability reported suspicions. However, this mechanism is also inhibited. In a context of inadequate diagnostic tools, comorbidities and practices of polypharmacy, patients, healthcare workers and retailers all find it difficult to judge a medicine’s effectiveness. They also face very real economic and social barriers to reporting, with retailers fearing damage to their reputation and business, while other whistle-blowers may not want to harm someone’s business and may also expect retribution.

Second, we find that regulators’ ability and willingness to investigate and sanction allegations is inhibited by difficulties in cooperating with other agencies in order to collect evidence. This could potentially be solved by more clearly specifying strategies and allocating responsibilities. But there are also more pragmatic hindrances and ethical quandaries. Regulators must manage relationships with retailers over many years, and hence seek to balance tough enforcement against the need to elicit compliance. In rural areas, tough enforcement against a non-compliant retailer might lead to an area’s only medicines outlet closing down, with serious health implications for the wider community.

Third, it seems unlikely that the current regulatory system has much effect in deterring misconduct or non-compliance. Our evidence suggests regulatory violations are often ignored and, even if exposed, are unlikely to be sanctioned. However, our findings also cast doubt on the extent to which we should characterise weak regulation of SF medicines as a problem of ‘misconduct’. Certainly, for regulators, there are a number of reasons to understand gaps in identifying, reporting, investigating and enforcing compliance more as inevitable consequences of weak capacity and difficult operating conditions.

## Conclusion

In summary, this study has shown that SF medicines are a particularly complex policy area in which to roll out a global monitoring and reporting mechanism such as the GSMS, raising serious questions about their potential effectiveness without very substantial investment in capacity of local regulatory authorities. We should reiterate that these problems are not unique to Tanzania; indeed (as noted earlier), Tanzania’s regulatory authorities are widely regarded to be among the best-functioning in sub-Saharan Africa. The challenges we have described here are likely to be the tip of the iceberg in the context of the African region as a whole.

This study has certain limitations. It is based on interviews with regulators and regulatees and relies on their own understandings of the problem and what they were willing to share with the research team. In such a sensitive area, and in a context where government was becoming increasingly tough on misconduct and corruption, it is possible that our research under-estimates the role of deliberate subversion of regulatory processes. However, in a context of low regulatory capacity, it is also difficult to distinguish the more or less innocuous causes of implementation gaps.

The study is also cross-sectional, making it difficult to disentangle causal effects, particularly when dealing with supply chains of medicines that may be long, complex and opaque, with no single actor having oversight of the whole. Future research could seek to understand how complex relationships between regulators, commercial actors, health-workers and consumers of medicines unfold over time as regulatory and reporting regimes change.

## Data Availability

Data are available upon request. Data are not publicly available but may be made available on request from heather.hamill@sociology.ox.ac.uk
